# Is Private Health Care the Answer to the Health Problems of the World's Poor?

**DOI:** 10.1371/journal.pmed.0050233

**Published:** 2008-11-25

**Authors:** Kara Hanson, Lucy Gilson, Catherine Goodman, Anne Mills, Richard Smith, Richard Feachem, Neelam Sekhri Feachem, Tracey Perez Koehlmoos, Heather Kinlaw

## Abstract

Background to the debate: The global burden of disease falls disproportionately upon the world's low-income countries, which are often struggling with weak health systems. Both the public and private sector deliver health care in these countries, but the appropriate role for each of these sectors in health system strengthening remains controversial. This debate examines whether the private sector should step up its involvement in the health systems of low-income countries.

## Abstract 2

This Debate examines whether the private sector should step up its involvement in the health systems of low-income countries.

## Viewpoint by Kara Hanson, Lucy Gilson, Catherine Goodman, and Anne Mills: There Is No Alternative to Strengthening the Public Role in the Health System

Is private health care the answer for the world's poor? Our starting point is that there are no strong grounds for assuming the superiority of either public or private health care. Theory tells us that it is not whether a health facility is publicly or privately owned that determines health provider performance. Instead, what influences performance is the nature of incentives that providers face and the quality of management and oversight. Theory does, however, suggest that the profit-making incentive dominant in much of the private sector is likely to be problematic for health care. Indeed, the reasons why private health care markets fail can be found in any introductory health economics text: (1) key preventive and public health services that produce external benefits (for example, prevention of spread of communicable disease or reduction in spread of antimicrobial resistance) will tend to be under-provided by private markets because these additional benefits are not valued in the market transaction; and (2) the patient's lack of technical knowledge, and the role of health providers in directing patient care, leave patients vulnerable to low-quality treatment, excessive use of diagnostics, and over-prescription.

However, empirical evidence on the performance of the two sectors also gives no clear guide to policy. In many settings, governments fail to provide health workers with incentives for good performance, offering health services of inadequate quality and allowing working environments to persist that engender uncaring and unresponsive attitudes amongst providers. Counter-intuitively, the public sector may even be inequitable, with some public services, especially at secondary and tertiary levels, disproportionately used by urban middle classes. At the same time, the private sector cannot be conceptualised as a single entity as there is a vast range of private providers operating throughout the low- and middle-income world. Private health services range from sophisticated inpatient facilities delivering advanced medical care of the highest international standard, through to the individual practices of doctors, nurses, and midwives, sometimes working in parallel with their public practice, and to unqualified peddlers of drugs from market stalls. What evidence there is suggests that poor people are more likely to use the lower-quality, highly dispersed, and fragmented end of this spectrum.

There is some limited evidence that the services of private providers can be improved through targeted quality improvement approaches [1]. But such projects have mostly operated at a relatively small scale, and have yet to demonstrate their ability to deliver improved population health. And such approaches have tended to engage with those parts of the private sector that are the easiest to organise—for example, those represented by recognised professional organisations—and less often with the informal, low-cost sources of care that provide the bulk of care to the poorest. The evidence reviewed in an International Finance Corporation publication on the private health sector in Africa supports the conclusion that existing initiatives have been limited in scale and focused on the more formal private health care sector [2].

Moreover, even where private services are low cost, they are not necessarily affordable. The evidence is clear that even short bouts of illness can have a catastrophic impact on welfare when households are very poor, threatening to undermine their livelihoods, and this impact is compounded in the case of chronic illness [3]. How health care is paid for is therefore crucial in assessing health care performance. But there is no evidence that private risk sharing schemes such as commercial insurance can reach the poorest groups, and the limited and seasonal cash income of these groups is likely to hamper attempts to include them in such schemes. While there are a few notable exceptions (such as Rwanda, where external resources have been used to expand insurance coverage), the reach of community-based insurance schemes has been very modest. There is therefore no alternative to strengthening the public role in financing health care, which can help achieve protection both against the cost of care and also against loss of income caused by illness.

Is there then scope for private providers to be paid through public financing? This arrangement requires some form of contract to act as a mechanism to (1) transfer public funds to private providers, (2) determine which health services should be provided by the contractor, and (3) monitor performance. The still-limited evidence on such arrangements includes interesting examples of contracts between the public sector and private non-governmental organisations in fragile states, such as Afghanistan [4]. But these, and wider contracting experiences, all point to the significant transactions costs of such arrangements and the need for strong and capable contracting units within health ministries [5].

Responding to the health needs of the poorest will require a major scale-up of coverage of good-quality primary care, referral to first-level hospital care, and mechanisms to protect poor households from catastrophic health care payments. But context will influence what role private providers play: in some environments, ensuring quality care through private provision may prove effective in reaching the poorest. In other settings, it may be more appropriate to focus on improving the way that public providers operate, by building supply chains, strengthening incentives to support good performance, and improving the quality of supervision and performance management. And in every context where private providers operate, governments need to oversee and regulate the health sector as a whole, including both public and private providers.

Identifying the appropriate roles for public and private health care sectors is challenging, and many questions remain about how best this can be achieved. What are the key capacities needed by governments to oversee the health sector? How do these vary by context, particularly in the face of uneven state capacity? And what forms of intervention are most effective at improving the performance of providers, both public and private? Building stronger health systems will require more innovation, more learning-by-doing, and more careful evaluation to understand what works and why, before it will be possible for countries to reach a firm conclusion about what range of solutions offer the most promise for the world's poor.

## Viewpoint by Richard Smith, Richard Feachem, Neelam Sekhri Feachem, Tracey Perez Koehlmoos, and Heather Kinlaw: We Must Engage the Private Sector to Improve Health Care in Low-Income Countries

In low-income countries today the private sector is a significant actor in health care—just as it is in high-income countries. Here we define the private sector as everything that is not the public sector, including non-governmental and faith-based organisations, social enterprises, for-profit companies, and a host of individual private providers in the formal and informal sectors. The private sector's role in health care should be strengthened and more closely aligned with the public interest. Indeed, a question recently posed by Anne Mills and colleagues is not how governments can finance and provide all health services, but instead how can private sector activities “be influenced so that they can help meet national objectives?” [6]. In recent years, there has been growing agreement in the international community that addressing health needs in developing countries and achieving the Millennium Development Goals requires that governments and donors actively engage with the private sector [7,8]. To have a national impact, true partnerships between governments and the private sector must be created that go well beyond the contracting out of basic services such as laundry or housekeeping to private companies.

Being pro–private sector does not imply being anti–public sector. The core of our argument is that, with a complicated problem such as improving health care under constrained resources, two heads are better than one. The public and private sectors have different strengths and weaknesses, and a judicious blending of the two can produce optimal results. Indeed, there is no health system that is entirely public or private [9]. Even in Britain, a country with an exceptionally high proportion of public spending, 13% of the health care spending in 2006 was private [10].

Significant amounts of public expenditure also go to the private sector in Britain and elsewhere. No “public” system produces its own drugs or equipment, and increasingly, governments in low- and middle-income countries are turning to the private sector to improve quality and deliver value for money. In China, Egypt, Lesotho, Mexico, Papua New Guinea, and South Africa, policy makers are partnering with the private sector to build infrastructure, provide staff and training, raise quality, improve productivity, undertake social marketing, enhance procurement, and much more [11,12].

The reality is that in most low-income countries, most people receive most of their care from the broadly defined private sector. About 60% of the US$16.7 billion spent on health in sub-Saharan Africa in 2005 was private, most of it out-of-pocket spending by individuals, and about half of this went to private providers [2]. This spending on the private sector is particularly pronounced in the lowest-income countries in sub-Saharan Africa [8]. In Afghanistan and India, about 80% of care is provided by the private sector [11]. Often it is the poorest who are most likely to use private providers: in South Asia, 80% of children in the lowest income quintile who have acute respiratory conditions and are brought for care use a private provider [13]; while in Africa about 50% of those who seek care outside the home go to private providers [14].

In some low- and middle-income countries, limited mechanisms already exist for engaging the private sector in accreditation, contracting, training, and the area of social franchising, which seeks to replicate the success of commercial franchising but for a social benefit [15].

Despite these experiences, systematic evidence on the effectiveness of encouraging the private sector in developing countries is sparse—because stewardship of the private sector may be weak, programmes may not have been evaluated, and results may not be published. Although many articles have emerged recently on private sector approaches, particularly on contracting and social franchising [1,16–21], it is hard to reach a confident conclusion from these studies. Nevertheless, there is undoubtedly widespread enthusiasm for social franchising and many are calling for public schemes to incorporate private providers to increase efficiency and equity [22,23].

Edith Patouillard and colleagues conducted a systematic review of whether the for-profit sector, both formal and informal, could help improve health care for the poor. They found 52 evaluated interventions and concluded that despite limited evidence, “many interventions have worked successfully in poor communities” [1].

Loevinsohn and Harding reviewed ten studies in which public authorities in developing countries had contracted with private organisations [24]. All ten found that use of the private sector produced positive results, and importantly, the more rigorous the study the more positive the results. The review showed that private contractors can operate on a large scale, be more cost effective than government-provided services, and increase coverage in poor and remote areas. In six studies that directly compared private with public provision, all found that the private sector did better on multiple measures of quality and coverage. Contracting to the private sector for immunisation services in Cambodia, for example, increased coverage by 40% compared with only a 19% increase using public providers. Also in Cambodia, when agreements with private providers specifically included targets for reaching the poor, equity was improved.

The current health systems “crisis” in low-income countries results from decades of neglect in the areas of finance, service delivery, and infrastructure. Many countries, and the donors that have supported them, have tried to address these challenges through an implicit policy of creating a public sector monopoly, ignoring the large and growing private sector gorilla in the room. Some countries are now exploring pluralistic models that partner with the private sector to serve public policy goals. These models should be encouraged and supported. Improving health care for the world's poor means harnessing everyone's capacity, not just that of governments.

## Hanson and Colleagues' Response to Smith and Colleagues' Viewpoint

Richard Smith and colleagues are forceful advocates for a greater role for the private sector in the health systems of low-income countries. Unfortunately, as they also recognise, the evidence to support their position is limited. In an era where national governments and donors are encouraged to embrace evidence-based policy making, blanket exhortations to harness the capacity of the private sector are unhelpful.

First, Smith and colleagues pay insufficient attention to the diversity of the private sector in developing countries. They cite data on sources of care from World Bank reports [13], but such data usually fail to capture this diversity, presenting results for all private sources together. Different types of providers offer different opportunities and threats for public health, and policies and interventions must be tailored to the provider type and context. For example, Smith and colleagues use evidence presented in the review by Loevinsohn and Harding [24] to advocate the use of private contractors, but fail to mention that many of the interventions cited in that paper involved non-governmental organisations. The lessons for working with private commercial providers are therefore unclear.

Second, Smith and colleagues place considerable weight on the proportion of private spending in total health financing. However, this is an imperfect measure of the size of the private sector. For instance, significant amounts of out-of-pocket spending go to public providers, through official user fees or informal charges, or go to purchase drugs and other inputs that are in short supply in public facilities. Moreover, high rates of out-of-pocket spending are a symptom of malaise in the health system rather than something to build on. There is abundant evidence of the potentially catastrophic impact of such payments on poor households [25]. Replacing out-of-pocket spending with private health insurance can bring its own problems in the form of coverage gaps and inefficiency. It is widely recognised that a strong public role in health spending (whether through payroll or general taxes) is essential for health systems to protect the poor [26]. Systems with the strongest state role have been shown to be the most equitable [27,28], and achieve better aggregate health outcomes [29].

Third, it is not true to say that governments and donors have completely ignored the private sector—the United States Agency for International Development, for example, has supported private sector provision initiatives since the mid-1980s [30]—but more attention to understanding the scale, scope, and effectiveness of this sector is certainly needed. The lack of evaluation of private sector initiatives is acute. For instance, in their systematic review Patouillard and colleagues identified no data on the impact of social franchising in low-income settings on technical quality or health outcomes [1]—so any “widespread enthusiasm” for it is hardly warranted. Smith and colleagues suggest that responsibility for this failure to evaluate interventions lies with the government stewards of the sector, but most private sector interventions have been donor supported. The global public health community must commit to developing a strong evidence base on private sector engagement so that future debates can be grounded in better understanding.

## Smith and Colleagues' Response to Hanson and Colleagues' Viewpoint

These two Viewpoints agree much more than they disagree. Both agree that the public sector cannot be ignored and both agree that there is a role for the private sector in improving the health of the world's poorest. The disagreement is about emphasis. We believe that many countries will benefit more from harnessing the energy of the private sector rather than continuing to invest solely or mainly in the public sector.

Five decades of attempts by governments in low-income countries to build state monopolies in health care have failed miserably in most of the poorest countries. Financing through general taxation is inadequate, and donor contributions are volatile and unreliable. Infrastructure has been allowed to decay, and service provision is often inequitable and of low quality [14,31–33]. Hanson and colleagues acknowledge these shortcomings. Our experience of working with health systems across the world is that the complexity of health care plus the bureaucracy and politicisation of government creates a lethal combination [8,34,35].

Hanson and colleagues mislead readers by repeating the familiar story of market failures. It is because of these inefficiencies that we are arguing for public–private partnerships, rather than simply the growth of an unregulated private sector.

We agree with Hanson and colleagues that more research is needed on the role of the private sector. But we have shown that there is more research than they may have recognised, and—importantly—this research points in the same direction, showing that the private sector can help the world's poorest. The absence of evidence is not, however, the same as evidence of absence of effect.

Policy makers and thought leaders in low- and middle-income countries, confronted with continuing failures in the public sector, growing evidence of the effectiveness of the private sector, and energetic non-state organisations, are already working to harness the power of the private sector to achieve better health care for all. Evaluation will be crucial, but the most important research question is not “Can the private sector help?” but “How can public–private partnerships be made most effective and equitable?”

As Hanson and colleagues rightly urge, we must innovate and learn by doing. In well-structured public–private partnerships, the private partners are fully accountable for the delivery of specified services and outcomes, and arrangements for financial rewards and penalties require that there is rigorous measurement of process and outcomes. None of this is true in a public system. A poor woman with an obstetric emergency in a rural area of a low-income country is likely to die. Her death and its cause go unrecorded. No inquiry is made about this preventable loss of life. No one is held accountable. No question is asked in parliament. Her death is a silent tragedy. The private sector can help us do better.
